# Atypical Symptoms Following Concussion: A Comprehensive Review of Functional Deficits

**DOI:** 10.1093/arclin/acaf051

**Published:** 2025-06-09

**Authors:** Anthony E Bishay, Natasha C Hughes, Avi N Albert, John E Dugan, Nick De Oliveira, Kristen L Williams, Scott L Zuckerman, Douglas P Terry

**Affiliations:** Vanderbilt University School of Medicine, Nashville, TN, USA; Vanderbilt University School of Medicine, Nashville, TN, USA; Meharry Medical College, Nashville TN, USA; Vanderbilt Sports Concussion Center, Vanderbilt University Medical Center, Nashville, TN, USA; Vanderbilt University School of Medicine, Nashville, TN, USA; Vanderbilt Sports Concussion Center, Vanderbilt University Medical Center, Nashville, TN, USA; Department of Neurological Surgery, Vanderbilt University Medical Center, Nashville, TN, USA; Vanderbilt Sports Concussion Center, Vanderbilt University Medical Center, Nashville, TN, USA; Department of Neurological Surgery, Vanderbilt University Medical Center, Nashville, TN, USA; Vanderbilt Sports Concussion Center, Vanderbilt University Medical Center, Nashville, TN, USA; Department of Neurological Surgery, Vanderbilt University Medical Center, Nashville, TN, USA

**Keywords:** Assessment, Emotions/emotional processing, Executive functions, Fluency (verbal/nonverbal), Language and language disorders, Movement disorders

## Abstract

**Objective:**

While most concussions present with common symptoms, some patients experience atypical manifestations that challenge diagnosis and management. This review synthesizes studies reporting atypical post-concussive symptoms with individual patient-level data, focusing on functional neurologic disorders.

**Methods:**

A systematic review was conducted across PubMed, EMBASE, and Cochrane Library databases (01/2000–08/2023). Inclusion criteria were: (1) study participants with concussion or mild traumatic brain injury, (2) trauma and/or sport-related injury, and (3) atypical symptoms without observable imaging findings. Atypical symptoms were defined as neurological symptoms not fully explained by traditional neuroanatomical or neuropathological correlates. Four independent reviewers screened titles, abstracts, and full texts. Data extraction included patient demographics, symptom characteristics, diagnostic methods, treatments, and outcomes.

**Results:**

Of the 4725 screened studies, 15 met inclusion criteria (2000–2022). Studies originated from five countries, with 8 (53.3%) from the United States. All studies were case reports (*n* = 12, 80.0%) or case series (*n* = 3, 20.0%). The review identified atypical symptoms across five domains: speech disorders (*n* = 6 studies, primarily new-onset stuttering), psychiatric alterations (*n* = 4 studies, including dissociative symptoms, Ganser syndrome, and psychotic features), visual changes (*n* = 1 study), hearing/vestibular disturbances (*n* = 2 studies), and gait abnormalities (*n* = 2 studies). Recovery patterns varied widely, ranging from complete resolution within weeks to persistent symptoms over several years.

**Conclusion:**

While most patients eventually improved with targeted interventions like speech therapy, psychiatric care, or physical therapy, recovery trajectories varied significantly. Larger prospective studies are needed to determine true incidence rates and establish evidence-based treatment protocols.

## INTRODUCTION

Concussions typically present with a common set of symptoms, often including headache, dizziness, visual disturbances, and are often divided into somatic symptoms, cognitive difficulties, emotional symptoms, and sleep difficulties (Tator, 2013). However, it is increasingly recognized that a significant number of patients also experience atypical symptoms following a concussion that likely are not related to the neurological impact of the head injury itself (Picon et al., 2021; Polich et al., 2024). These less common manifestations, often falling under the umbrella of functional neurologic disorders (FNDs), can be perplexing for both patients and healthcare providers, as they may not align with the expected post-concussive presentation (Polich et al., 2024). These atypical symptoms can affect various systems, including speech difficulties (Binder et al., 2012; Cherry & Gordon, 2017; Robertson & Diaz, 2020; Shah & Essad, 2019; Toldi & Jones, 2021; Yeoh et al., 2006), psychiatric symptoms (Ashish et al., 2017; Dalfen & Anthony, 2000; Diehl, 2009; Mackenzie, 2000), visual disturbances (2022 AMSSM Case Podium Presentations, 2022; Foutch, 2015; Lin & Yen, 2020), hearing abnormalities (Rotenberg et al., 2005; Wong & Abdul Kadir, 2015), and gait irregularities (Cowley et al., 2016; Wasylynko, 2017). The presence of these diverse and often unexpected symptoms can complicate diagnosis, treatment, and recovery, highlighting the need for a more comprehensive understanding of these rare post-concussion sequelae.

Atypical symptoms following concussions often manifest as complex FNDs. Consider the case of stuttering following a concussion, a phenomenon that can emerge without any detectable structural or neurological cause (Cherry & Gordon, 2017). Patients may find themselves struggling with fluent speech days or weeks after their injury, despite no prior history of a speech disorder, though those with a developmental history of stuttering or pre-existing psychiatric conditions may be at increased risk (Lundgren et al., 2010). This symptom typically follows an unpredictable course, often resolving spontaneously within weeks to months, yet leaves both patients and clinicians curious about its origin and management (Robertson & Diaz, 2020; Toldi & Jones, 2021). Similarly, functional gait disorders can dramatically affect a patient’s mobility and independence. An individual who previously walked without difficulty may suddenly exhibit an unusual, inconsistent gait pattern that defies physiological explanations. These symptoms can fluctuate in severity and persist for months (Cowley et al., 2016; Wasylynko, 2017). While many patients see gradual improvement with rehabilitation and psychological support, some may experience long-term difficulties (Wasylynko, 2017). The complex nature of these symptoms, coupled with their potential for significant impact on daily life, underscores the complexity of post-concussive care. However, there has been a very limited effort to amalgamate these symptoms into syndromal categories, leaving clinicians with fragmented information when encountering such cases.

The lack of comprehensive resources on atypical symptoms following concussion presents a significant challenge for healthcare practitioners. When encountering patients with these unusual manifestations, clinicians often find themselves with little empirical evidence to guide their approach. This knowledge gap can lead to uncertainty in diagnosis, treatment planning, and discussions about prognosis. Consequently, the objective of the current systematic review was to identify and synthesize studies reporting atypical post-concussion symptoms using granular, patient-level data to better equip practitioners with the knowledge needed to recognize, discuss, and manage these challenging cases.

## MATERIALS AND METHODS

### Search criteria and study selection

A comprehensive literature review was conducted to investigate the range of functional and atypical symptoms following concussion. The search terms included two primary elements: (1) concussion-related terms (“concussion,” “mild traumatic brain injury,” “post-concussion syndrome”) and (2) psychogenic-related terms (e.g., “functional neurological disorder,” “atypical symptoms,” “psychogenic,” “medically unexplained,” “somatic;” see Supplementary File). Multiple databases, including PubMed, EMBASE, and Cochrane Library, were searched from January 2000 to August 2023. The year 2000 was chosen as the baseline year for this study as it marks the beginning of increased research focus on mild traumatic brain injury and its long-term effects (Long et al., 2024; Tang et al., 2022).

For the purposes of this review, atypical symptoms or FNDs were defined as neurological symptoms that are not fully explained by traditional neuroanatomical or neuropathological correlates, in keeping with prior studies (Espay et al., 2018). These symptoms show clinical features inconsistent and not recognized following concussion and may fluctuate in severity or nature over time or with attention. They are not intentionally produced or feigned, cause significant distress or functional impairment, and cannot be attributed to another medical or psychiatric disorder. These symptoms may include, but are not limited to, abnormal movements, sensory disturbances, speech difficulties, gait problems, and cognitive complaints that are disproportionate to or inconsistent with the expected sequelae of a typical concussion.

### Inclusion criteria

Original studies that investigated atypical symptoms following concussion, with granular, patient-specific data, were considered for inclusion. Letters to the editor, commentaries, and editorial articles were excluded from review. The search process involved screening the title, abstract, and full-text article. Studies were included only if the patient presented with ongoing atypical or functional symptom presentation, and if no findings were observed across any imaging modalities or clinical examinations/tests. Only studies reporting individual patient-level data with detailed clinical information were included in this review. Studies presenting exclusively group-level statistics or aggregated data without granular case-specific details on symptom presentation, diagnostic timeline, and treatment outcomes were excluded. Studies that reported cranial nerve palsies, even if no findings were observed on imaging, were excluded due to these symptoms often having a clear neuroanatomical basis and are not typically classified as FND.

**Fig. 1:**
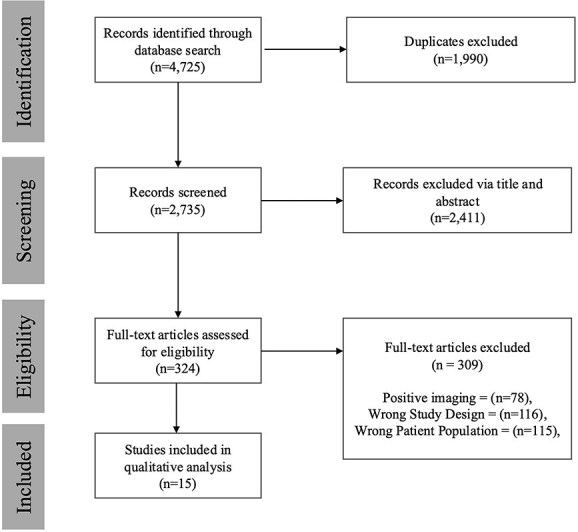
PRISMA flow diagram depicting study criteria.

While studies from all countries were considered, an English version of the article was required for inclusion. The screening process was conducted by four independent reviewers using Covidence (Covidence, n.d.), a web-based software platform for managing systematic reviews. For a study to be either included or excluded, two reviewers needed to agree. In cases of disagreement, a third reviewer was brought in to help reach a consensus. A PRISMA flow diagram detailing the search and selection process is provided in Fig.  1.

### Data extraction

Following the selection of studies using Covidence, data were manually extracted from the included articles by the same authors who conducted the screening. The following information was collected for each study: study characteristics (title, authors, publication year, journal, and study design); sample information (total sample size, number of patients with mild traumatic brain injury or concussion with functional symptoms, number of controls if applicable, age, gender distribution, and race if reported); clinical data (time after concussion, medical and psychiatric history, previously prescribed medications, number of prior concussions, and mechanism of injury); diagnostic information (lab findings, head imaging results); symptom details (functional neurological/somatic symptoms, symptom grouping); assessment tools (scales used and their results); and recovery and treatment information (recovery measurements, time to recovery, treatments used, number of sessions, total length of treatment, time to last follow-up, functional symptom resolution, and any persistent symptoms). For studies that provided detailed information about individual patients, each case was assigned a unique identifier number to facilitate individual case analysis.

### Data synthesis and analysis

The extracted data were synthesized qualitatively to identify patterns and trends in the presentation, course, and management of atypical and functional symptoms following concussion. Symptoms were categorized into major groupings to facilitate analysis and presentation of findings. Due to the heterogeneous nature of the studies and reported outcomes, a meta-analysis was not performed. Instead, a narrative synthesis of the findings was conducted, focusing on describing the range of atypical symptoms, their prevalence, course, and reported treatment outcomes.

## RESULTS

### Article characteristics

Of the 4725 initially screened studies, 15 met inclusion criteria (Fig.  1), with publication years spanning 2000–2021. Studies originated from 6 different countries, with 7 (43.8%) from the United States. All studies were either case reports (*n* = 12, 80.0%) or case series (*n* = 3, 20.0%). A total of two studies (13.3%) described compensation-seeking status of patients (Binder et al., 2012; Diehl, 2009), whereas only one study (6.7%) described performance validity test results (Binder et al., 2012). All results, including age of patients, symptoms, and outcomes are summarized in Table 1. Specific case descriptions per FND domain are synthesized earlier.

**Table 1:** Detailed study characteristics

**Symptom grouping**	**Author and year**	**Study design**	**Total sample size**	**Age, gender**	**Time after concussion**	**Medical history/diagnoses**	**Psychiatric history/diagnoses**	**Number of prior concussions**	**Mechanism of injury**	**Imaging**	**Functional neurological/somatic symptoms**	**Treatments used**	**Total length of treatment**	**Time to last follow-up**	**Functional symptom resolution (yes/no)**	**Persistent symptoms**
Speech	Robertson 2020	Case Report	1	16, F	1 d	None	None	0	Fell backwards during soccer match	MRI	Stuttering, including while singing	Speech therapy	6 mo	6 mo	Yes	No
Speech	Yeoh 2006	Case Report	1	12, M	Immediate	None	None	0	Fell prone from his skateboard, resulting in a mild head injury	CT	Stutter	None	NS	4 wk	Yes	No
Speech	Toldi 2021	Case Report	1	15, M	3 d	None	None	0	Helmet-to-helmet collision during a football game	CT, MRI	Stutter	Vestibular physical therapy, behavioral health sessions for anxiety and depression, speech therapy	NS	6 wk	Yes	No
Speech	Shah 2019	Case Series	3	14, F	NS	None	Anxiety	NS	Volleyball	NS	Nonphysiologic stutter and atasia-abasia	CBT	NS	NS	NS	No
				16, F			Depression	1	Soccer		Slurred speech and stuttering	Partial day program with psychiatry and intensive speech therapy			Yes	
				18, M			None	NS	NS		Catatonia with echolalia	Diazepam			Partial improvement	
Speech	Cherry 2017	Case Report	1	10, M	CT, MRI	None	None	NS	Fall	CT	Seizures	Antiepileptic drugs, oral steroids	2 y	2 y	No	Yes
Speech	Binder 2012	Case Series	3	40, F	11 d	None	None	0	MVA	MRI, EEG	Stuttering, “baby talk,” astasia-abasia, sensory impairment	None	None	31 mo	NS	NS
				41, M	3 mo				Object fell on head	None	Cognitive difficulty, speech-language, emotional	None	None	NS		
				37, F	2 wk				Fell off horse	CT, MRI	Stuttering, functional dependence, chronic pain, cognitive difficulties	Speech therapy	40 mo	40 mo		
Psychiatric	Diehl 2009	Case Report	1	50, F	Immediate	None	Borderline personality disorder	0	Head striking floor secondary to syncopal episode	CT	Dissociative symptoms	Dialectical behavioral therapy	Weekly for multiple months	Ongoing at the time of publication	No	Yes
Psychiatric	Dalfen 2000	Case Series	4	35, F	0 d	None	None	NS	MVA	CT	Handedness change, inappropriate laughter, absurd answers	Unknown	Unknown	Unknown	Unknown	Unknown
				42, F	“Few days”				Large frame hit her head	MRI/SPECT	Writing backwards, halting speech, bizarre cognitive answers					
				26, M	0 d				Kicked in head at concert	CT	Inappropriate affect, approximate answers					
				38, F	“Few days”				MVA	No	Labile affect, approximate answers					
Psychiatric	Ross 2000	Case Report	1	56, F	7 d	Partial thyroidectomy, hysterectomy	Depression	NS	Door to head	CT, EEG	Word-finding difficulties, excessive fatigue, dizziness, tingling in arms and legs	NS	Unknown	3 y	No	Yes
Psychiatric	Ashish 2017	Case Report	1	14, M	7 wk	None	None	2	Football	MRI	Hallucinations 3 wk following injury	Acceptance and commitment therapy, family therapy, vestibular-ocular physical therapy	28 wk	18 mo	Yes	No
Visual	Foutch 2015	Case Report	1	36, F	3 mo	None	None	0	MVA	No	Unilateral anopia	Emotional expression	–	–	Yes	Unknown
Hearing/Vestibular	Rotenberg 2005	Case Report	1	11, M	24 h	None	None	0	mild blunt right-head injury	CT	Acute right-sided total hearing loss; conversion disorder	None	NS	1 wk	Yes	No
Hearing/Vestibular	Wong 2015	Case Report	1	33, F	NS	None	None	0	minor head injury during a roller coaster ride	MRI	Bilateral vestibulopathy, chronic subjective dizziness, spondyloarthropathy	Betahistine, sulfasalazine, oral prednisolone, benzodiazepine	3 mo	4 mo	Yes	Yes, vertigo
Gait	Wasylynko 2017	Case Report	1	10, M	5 d	None	None	NS	Hit head while swimming	MRI	Lower limb muscle weakness and ataxia, foot numbness, difficulty walking	Chiropractic manipulative therapy, physiotherapist, occupational therapist, psychologist	1.5 y	1.5 y	Yes	No
Gait	Cowley 2016	Case Report	1	52, M	NS	None	None	NS	Golf club injury to the head	CT	Right-sided hemiparesis	Saline	NS	18 mo	Yes	No

*Note*: CT, computed tomography; d, days; EEG, electroencephalogram; F, female; h, hours; M, male; mo, months; MRI, magnetic resonance imaging; NS, not stated; wk, week(s); y, years.

### Speech changes

Six studies included in our review reported patients with abnormal speech pathology following concussion. Robertson and Diaz (2020) described the case of a 16-year-old girl with no reported prior medical history who experienced stuttering after hitting the back of her head on the ground during a soccer match. The injury occurred when she tripped, causing her to feel dazed but without loss of consciousness. Initially, she reported blurry vision and headache, which resolved by the following day. However, upon waking the next morning, she developed significant stuttering during both regular speech and singing. Neurological examination was otherwise normal, and cranial imaging showed no abnormalities. Treatment involved sessions with a speech pathologist. The patient’s symptoms began to improve after 8 weeks of therapy, with near-complete resolution at 12 weeks. At the 6-month follow-up, all residual speech problems had fully resolved.

Another case report by Yeoh and colleagues (2006) described the case of a 12-year-old boy with no reported prior medical history who experienced acute stuttering immediately after falling from his skateboard. The patient was unconscious for less than a minute and had less than 20 min of anterograde amnesia. Initial symptoms included persistent headache, difficulty producing speech, marked incoordination, inability to ambulate independently, truncal ataxia, and positive Romberg test. No sensory deficits were noted. Diagnostic tests, including electrolyte levels, complete blood count, and intracranial imaging studies, were all normal. After he was hospitalized overnight, all symptoms resolved by the following morning. At the 4-week follow-up, the patient’s mother reported he was completely back to normal, with only concentration issues persisting for 1–2 weeks before subsiding.

Toldi and Jones (2021) reported a 15-year-old boy with no reported prior medical history who developed new-onset stuttering after a helmet-to-helmet hit during a football game. He continued playing until a severe headache triggered his removal from play. Three days post-injury, he presented to the clinic with symptoms including fogginess, dizziness, and headache. Head CT was negative, but the patient exhibited stuttering and mildly slowed cognition, including slowed processing speed and word-finding difficulties. Vestibular/ocular-motor screening was halted due to worsening symptoms. The patient scored 69/132 on the Post-Concussion Symptom Scale, with higher scores for dizziness, feeling “foggy,” and difficulty concentrating. Treatment involved vestibular physical therapy and behavioral therapy. The patient's symptoms, including stuttering, improved over time, with complete resolution of stuttering after one month. However, headache persisted. At the last follow-up, the patient reported significant improvement, though the stuttering returned when he was feeling excited or nervous. While instructed to continue prescribed therapies, the patient was lost to follow-up after this point.

Shah & Essad (2019) reported three cases of abnormal speech following concussion. The first case involved a 14-year-old girl with a history of prolonged concussion recovery due to anxiety. She experienced a volleyball-related concussion. Months later, she was struck by a tennis ball, an incident that did not cause a second concussion but was followed by the development of a non-physiologic stutter and astasia-abasia (i.e., inability to stand or walk). The patient’s speech problems resolved completely with cognitive behavioral therapy (CBT). The second case was a 16-year-old girl with a history of depression who sustained a concussion during a soccer match. She initially presented with slurred speech, which improved after 2 weeks but was replaced by new-onset stuttering. The stuttering improved with psychiatric medication adjustment but returned 2 years later after a second concussion. Further treatment with a partial day program involving psychiatry and intensive speech therapy led to improvement in symptoms. The third case involved an 18-year-old male wrestler who displayed catatonia with echolalia after sustaining a concussion. Treatment with diazepam resulted in partial improvement of symptoms, but the patient was unable to participate in CBT. No further follow-up was reported for this case.

A case report by Cherry & Gordon (2017) described a 12-year-old girl who experienced new-onset stuttering 24 hr after she hit the back of her head while walking down a snowy staircase. Her initial symptoms after the fall included headache, photophobia, and drowsiness. Her neurologic exam was otherwise normal. At presentation to a pediatric neurology concussion clinic 4 days after the fall, most of her symptoms had resolved except for her stuttering. She was then referred to speech pathology. By 2 weeks after the injury, her stuttering had completely resolved.

A case series by Binder and colleagues (2012) reported three patients with atypical psychiatric symptoms following concussion suggestive of somatoform disorders or malingering. The first patient, a 40-year-old female, suffered a motor vehicle accident and presented with stuttering, weakness in all four extremities, and “baby talk” with inconsistent language errors. The second patient, a 41-year-old male, experienced a work-related injury when an industrial roll of paper fell on his head, presenting with varying pressured or sluggish speech and inconsistent stuttering. The third patient, a 37-year-old woman, was injured in a horse jumping accident and developed speech difficulties. Initial symptoms for the first two patients were not specified, whereas the third patient developed symptoms the day after her injury. Diagnostic findings for the first two patients included implausible neuropsychological exam results resembling malingering. The third patient’s multiple CT scans, MRI, EEG, and carotid ultrasounds were normal. Treatment approaches varied: the first two patients had no reported treatment, whereas the third underwent speech therapy. Outcomes differed: the first patient’s symptoms persisted for at least 3 years, the second patient was lost to follow-up, and the third showed initial improvement but continued to exhibit peculiar speech 40 months post-injury. The authors concluded that the first two cases were likely malingering, whereas the third was attributed to a pre-existing somatoform disorder exacerbated by the injury and possibly influenced by an ongoing personal injury lawsuit.

*Section Summary*: Speech changes, particularly new-onset stuttering, can occur following concussion in both pediatric and adult patients, with most cases resolving within weeks to months with appropriate treatment like speech therapy. Symptoms typically develop either immediately after injury or within 24 hr, and often present alongside other common post-concussive symptoms like headache and dizziness.

### Psychiatric changes

Four studies identified various atypical psychiatric post-concussive symptoms, with dissociative symptoms being most reported. Diehl (2009) reported that a 50-year-old woman with a history of borderline personality disorder and major depressive disorder, including multiple hospitalizations and antidepressant trials, sustained a work-related injury. She fell and hit her head on a wooden table, losing consciousness. CT findings were negative, leading to a mild TBI diagnosis. Over the following 3 months, she experienced intermittent amnestic mental status changes, disrupted identity, memory loss, and altered self-perception. Neuropsychological testing revealed psychological distress, “inconsistent performance,” (Diehl, 2009) and potential intentional exaggeration of somatic complaints. The author attributed these atypical symptoms more to her pre-existing borderline personality disorder than to the mild TBI. Treatment consisted of three months of weekly dialectical behavioral therapy. However, her dissociative symptoms persisted without improvement in the ensuing years.

Additionally, another article by Dalfen and Anthony (2000) discussed four patients (35-year-old female, 42-year-old female, 26-year-old male, and 38-year-old female) who presented with Ganser syndrome (i.e., production of approximate answers to simple questions (Dieguez, 2018)) following head injuries. Only the 26-year-old male had prior psychiatric history of a fugue state. Two patients suffered motor vehicle accidents, one was injured by a large frame falling on their head, and one was kicked in the head at a rock concert. All patients exhibited atypical symptoms characterized by approximate incorrect answers to simple questions and math problems, such as calling a ball an umbrella or stating that 2 + 2 = 5. Three patients also displayed inappropriate affect, including inappropriate laughter or cheerfulness. These symptoms persisted for at least several months in all cases. Diagnostic findings were not specified. One patient was ultimately diagnosed with post-traumatic stress disorder, whereas two others reported intrusive recollections of their accidents but did not meet full PTSD criteria. Treatment approaches were not detailed. At the time of last follow-up, all patients still exhibited symptoms, and only one was seeking litigation. Unfortunately, long-term outcomes and symptom resolution were not reported.

One case report by Mackenzie (2000) discussed a 56-year-old woman with a history of severe depression, electroconvulsive therapy, and anxiety. She sustained a left temple injury against a door, resulting in a concussion. Initially dazed and disoriented with anterograde amnesia, she was admitted to the hospital with a GCS of 15 and discharged after 2 days. Her symptoms worsened over the next week, leading to re-admission due to inability to recall events and her name, unsteady gait, slow speech with word-finding difficulty, odd affect, and numbness and tingling in her extremities. EEG and CT head were normal, but neuropsychological testing revealed significant impairments in verbal fluency, recognition memory, and remote memory. The patient had recently moved to England for her fiancé, only to discover that he was living with someone else and ended the relationship. Her atypical symptoms included extensive amnesia, particularly for childhood and early adulthood. The authors suggested a combination of organic and functional causes, possibly related to dependent personality disorder. No specific treatment was reported. Despite multiple follow-ups over three years, the patient’s symptoms persisted without resolution, though treatment efforts were not reported by the author.

A case report by Ashish and colleagues (2017) identified a 14-year-old male football player with no prior psychiatric history who suffered his third mild TBI during practice. Three weeks post-injury, he developed visual and auditory hallucinations occurring several times daily, featuring negative and disparaging content such as a frightening figure saying, “you will not get better.” The patient reported moderate depression (Beck Depression Inventory = 27) and anxiety (Beck Anxiety Inventory = 23), including thoughts of self-harm. Diagnosis was not specified though psychosis and psychological disturbances were prominent. Treatment approach involved 11 sessions of Acceptance and Commitment Therapy and 7 sessions of family therapy to improve communication, as parents refused pharmacologic treatment. By the end of treatment, the patient’s self-rated anxiety decreased by 40%, and he returned to school after a 3-month absence. At 18-month follow-up, the patient reported complete resolution of psychotic symptoms with no hallucinations for over a year.

*Section Summary:* Post-concussive psychiatric symptoms can include dissociative symptoms, Ganser syndrome, and in rare cases psychotic features like hallucinations. Unlike most post-concussive symptoms, these psychiatric manifestations often show a more prolonged or chronic course, particularly in patients with pre-existing mental health conditions.

### Visual acuity changes

A total of one study described visual changes following head injury. A study by Foutch (2015) discusses the case of a 36-year-old Hispanic female with no significant medical history who experienced sudden right-eye blindness three months after sustaining a concussion from a motor vehicle collision. Her initial visual acuity was measured at 20/400 oculus dexter (OD) and 20/25+ oculus sinister (OS), with improvement to 20/50 OD using pinhole and binocular polarized testing. Comprehensive ophthalmologic examinations revealed no organic abnormalities. Follow-up assessments showed dense nasal hemianopia OS and blank visual field OD. The patient exhibited an atypical ability to perceive diplopia under various test conditions, indicating intact visual function in OD despite severe visual field loss. Emotional distress was identified as a contributing factor, as her visual acuity spontaneously improved to 20/20 in both eyes after crying and expressing personal emotional frustrations. A diagnosis of vision change disorder was considered. The patient was advised on symptom management and referred for neurological evaluation and behavioral management. Unfortunately, long-term follow-up was not available due to loss to follow-up.

*Section Summary:* While visual changes such as blurry or cloudy vision are common following concussion, atypical vison changes can rarely occur, with symptoms like sudden vision loss occurring weeks to months after injury. The case evidence suggests these symptoms may improve spontaneously, particularly when emotional factors are addressed, though limited long-term follow-up data exists.

### Hearing and vestibular changes

Two studies were found that documented unusual hearing or vestibular changes following minor head trauma. A case report by Rotenberg and colleagues (2005) describes an 11-year-old boy with no past medical or psychiatric history presented with sudden sensorineural hearing loss following mild head trauma. He experienced acute right-sided total hearing loss 24 hr after mild trauma to the right side of his head. Initially, the patient had no headache, nausea, photophonophobia, abnormal gait, loss of consciousness, tinnitus, or vertigo. CT scans showed no abnormalities, and auditory exams were normal except for a Weber test lateralized to the left. Audiograms revealed profound right-sided sensorineural hearing loss from 250 to 8,000 Hz with normal left-sided hearing. Additional relevant history included possible home stressors, withdrawn behavior at school, and a maternal history of conversion disorder. The patient was monitored and scheduled for a repeat audiogram. One week after discharge, his hearing spontaneously returned to normal after hitting his head on a school bus window. At follow-up, the patient reported mild dizziness and nausea without vomiting or eye-twitching. No specific treatment was mentioned, but the patient’s hearing fully recovered within a week of the initial presentation.

A case report by Wong & Abdul Kadir (2015) describes a 33-year-old female with no significant past medical or psychiatric history experienced multiple instances of vertigo after minor head trauma sustained during a roller coaster ride. Initial examination revealed no physical abnormalities, and brain MRIs showed no abnormalities. Further evaluation led to a diagnosis of bilateral peripheral vestibulopathy without hearing impairment. The patient was treated with betahistine, resulting in reduced vertigo attacks after 12 months, though dizziness persisted for an additional year. Approximately 2 years post injury, the patient developed autoimmune spondyloarthropathy. Treatment for spondyloarthropathy led to remission of the vertigo and dizziness, though these symptoms recurred 6 months later. A psychiatric assessment 3 years after initial presentation resulted in diagnoses of agoraphobia without panic attacks, hypersensitivity to environmental changes, and chronic subjective dizziness. The patient’s condition was successfully treated with a benzodiazepine. The case demonstrates an unusual progression of symptoms and their relationship to other developing conditions over a 3-year period.

*Section Summary:* Hearing and vestibular changes following concussion can range from temporary hearing loss to chronic vertigo, with symptoms showing variable courses. While these symptoms are common after concussion and typically have organic causes, functional disorders may be considered after ruling out other explanations when symptoms present atypically. These sensory disruptions may resolve spontaneously or require long-term management depending on their underlying mechanisms and associated conditions.

### Gait abnormalities

Two studies reported gait abnormalities following concussion. A case report by Wasylynko (2017) described a 10-year-old male with no past medical or psychiatric history who sustained a concussion while swimming. Immediately following injury, he was taken to the emergency department (ED) due to neck pain, dizziness, blurred vision, and an unsteady gait. The patient presented 5 days following injury due to lower limb muscle weakness and ataxia, foot numbness, and difficulty walking. No abnormalities were observed on MRI. The patient was referred to various specialists including a physiotherapist, occupational therapist, psychologist, and a chiropractor. The patient received chiropractic manipulative therapy 4 times in a 1 week, followed by consistent sessions once per month. Resolution of these symptoms occurred after 1.5 years of chiropractic manipulative therapy.

Another case report by Cowley and colleagues (2016) described a 52-year-old male with no past medical history who suffered golf-club trauma to the head. Immediately following injury, the patient appeared to have a 3-min generalized tonic–clonic seizure. Initial evaluation revealed a 13 Glasgow Coma Scale, with deficits only in eye movement and verbal commands, a dilated right pupil, numbness in the upper and lower extremities, and right-sided hemiparesis. The patient was started on hypertonic saline infusions for his closed-head injury and did not receive any other medication. No findings were observed on CT, such as generalized cerebral edema, though the patient did have a nasal bone fracture. An electroencephalogram was not performed. The patient experienced prolonged right-sided hemiparesis for 24 hr, which then began to improve with no additional treatment. The patient continued to receive maxillofacial care for 18 months following the injury.

*Section Summary:* Post-concussive gait abnormalities can present with varying severity and duration, from temporary hemiparesis to prolonged ataxia and muscle weakness. Recovery timeframes range from days to years, with treatment approaches varying from conservative management to long-term therapeutic interventions.

## DISCUSSION

This comprehensive review of atypical symptoms following concussion reveals a wide spectrum of unusual presentations across multiple domains. The findings encompass speech disorders, psychiatric changes, visual disturbances, hearing and vestibular abnormalities, and gait abnormalities. These atypical symptoms often present significant diagnostic and management challenges due to their unexpected nature and variable course. While some cases resolved spontaneously or with targeted interventions, others persisted for extended periods of time, highlighting the complexity of these sequelae. The review underscores the importance of considering both physiological and functional etiologies in the assessment and treatment of these atypical symptoms, as well as the need for a multidisciplinary approach to patient care.

### Symptom specific discussion

#### Speech changes

In six studies of 10 patients, several cases of atypical speech changes, primarily stuttering, were observed in adolescents and young adults following concussion. These symptoms typically emerged within 24 hr post-injury and varied in duration from days to months. Most cases resolved spontaneously or with targeted interventions, such as speech therapy, CBT, or medication adjustments. Some cases showed a relationship between emotional states (e.g., anxiety, excitement) and symptom exacerbation, suggesting a functional component. A multidisciplinary approach involving speech pathology, neurology, and psychiatry may be beneficial, with future research needed to identify predictors and develop evidence-based treatment protocols.

#### Psychiatric changes

In four studies of seven patients, a spectrum of atypical psychiatric symptoms, with dissociative symptoms predominating, including Ganser syndrome, extensive amnesia, and post-concussive psychosis were observed following concussion. These cases highlight the complex interplay between pre-existing psychiatric conditions, brain injury, and psychosocial factors. Recovery varied significantly, from complete resolution to persistent symptoms. Treatment approaches, including psychotherapy and family therapy, showed some success, but long-term follow-up data remain limited. Several patients, but not all, had a personal history of psychiatric disorders with unknown family history. Future research should focus on developing standardized assessment tools and investigating the role of pre-existing conditions in symptom development.

#### Visual changes

In one study of one patient, diverse visual symptoms, ranging from sudden blindness to transient vision loss were seen following concussion. The potential link between emotional factors and visual symptoms warrants further investigation, with long-term follow-up data notably lacking. Clinicians should be prepared for both physiological and functional visual disturbances, considering a multidisciplinary approach when necessary. Referral to ophthalmology is essential to rule out serious conditions, followed by reassurance and watchful waiting as appropriate, as some cases may resolve spontaneously, whereas others require more intensive intervention.

#### Hearing and vestibular changes

In two studies of two patients, atypical hearing and vestibular changes were presented, including sudden sensorineural hearing loss in a child and persistent vertigo in an adult. The spontaneous resolution of symptoms in both cases, particularly the child’s hearing loss resolving after another minor impact, suggests a functional component. Clinicians should consider both organic and functional etiologies when evaluating unusual auditory or vestibular symptoms. A multidisciplinary approach, including psychiatric assessment, may be beneficial in managing these cases, with further research needed to understand the underlying mechanisms.

#### Gait abnormalities

In two studies of two patients, the studies present two distinct cases of gait abnormalities following concussion, demonstrating the varied presentations and recovery trajectories of these symptoms. One case involved a child with ataxia and lower limb weakness persisting for 1.5 years, whereas the other described an adult with transient hemiparesis. These cases highlight the potential for both short-term and long-term gait disturbances, even without significant neuroimaging findings. A multidisciplinary treatment approach, involving physiotherapy, occupational therapy, and psychological support, is recommended. The prolonged pediatric recovery emphasizes the importance of long-term follow-up and persistent intervention.

### General discussion and future directions

The current review reveals the variability and multifaceted nature of atypical symptoms following concussion, highlighting a critical need for systematic research and patient-centered care. Despite low reporting rates—and likely low base rates of such atypical presentations—these symptoms, spanning speech, psychiatric, visual, auditory, and motor domains, demonstrate the heterogeneity of post-concussive experiences that extend beyond traditional neurological sequelae. These presentations are not indicators of permanent brain damage, but rather, indicate nuanced manifestations of the body’s adaptive response to injury, integrating physiological, psychological, and environmental factors.

Recent studies further substantiate these observations, revealing that patients with post-concussion functional disorders often present with high rates of baseline risk factors. While these studies were not included in our systematic review due to their group-level analysis, they provide important complementary insights into the complex nature of post-concussion functional disorders. Stubbs and colleagues (2020) highlights that somatic symptoms (including pain, sensory changes, and other clinical manifestations) are more prevalent after mild traumatic brain injury, suggesting a broader spectrum of post-concussive manifestations beyond specific functional disorders. Green and colleagues (2022) found that adolescents with somatization demonstrated more severe atypical neurological symptoms and increased healthcare utilization, whereas Polich and colleagues (2024) documented a wide range of functional neurological symptoms including gait disturbances, seizures, and speech abnormalities emerging either abruptly or gradually after concussion.

### Our approach

**Fig. 2:**
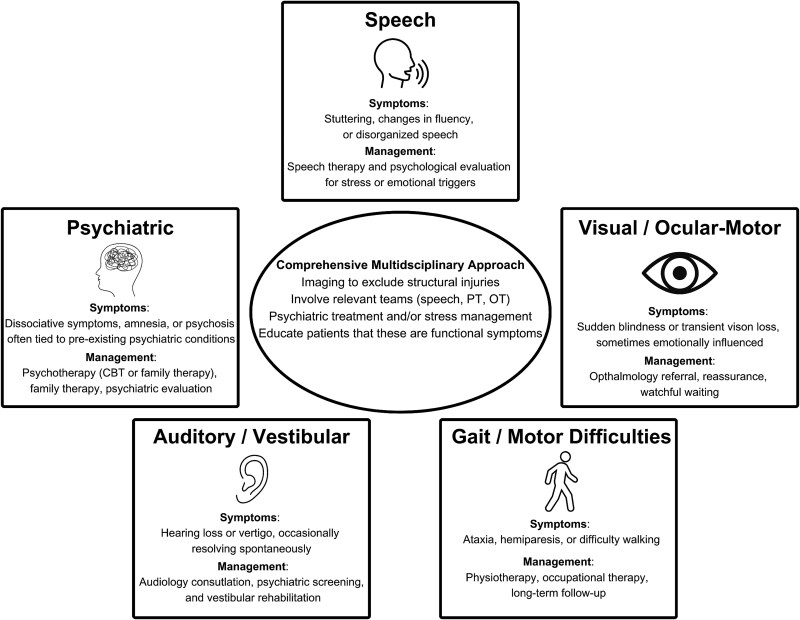
Atypical post-concussion symptoms and management.

At our institution, management of atypical symptoms following concussion involves a systematic and multidisciplinary strategy. Imaging is prioritized to confirm the absence of structural brain injury and rule out serious pathology. Following this, relevant specialists, including neurology, psychiatry, ophthalmology, speech therapy, and physical therapy, are engaged as needed to address the wide spectrum of symptoms. Patient education is a critical component, emphasizing that these symptoms do not represent a more severe brain injury or a worsening concussion but are often functional responses to trauma. Early identification of these symptom patterns allows for targeted interventions in order to optimize outcomes. Fig.  2 illustrates these domains as well as their corresponding management strategies.

### Limitations

This review has several limitations that should be considered when interpreting the results. First, many of the included studies are from earlier years, which means the imaging modalities used were less advanced than current technologies. This limitation in imaging sensitivity may have led to an underestimation of structural abnormalities. For example, a 2017 study reported the case of a 26-year-old female with tremor in her lower extremities (Jang & Lee, 2017). While T1, T2, and fluid-attenuated inversion recovery (FLAIR) imaging showed no findings, diffusion tensor imaging with affine multi-scale two-dimensional registration revealed traumatic axonal injury, highlighting how advanced imaging techniques can uncover previously undetectable abnormalities. The heterogeneity of the included studies in terms of patient populations, assessment methods, and follow-up periods makes it challenging to draw definitive conclusions about the nature and course of these atypical symptoms. The reliance on case reports and small case series limits the generalizability of the findings. Contrary to typical publication bias, there may be an underrepresentation of these atypical cases in the literature, as many journals are reluctant to accept case reports. This could lead to an underestimation of the occurrence of these unusual symptoms. As a result, these methodological constraints significantly impede our ability to systematically determine the true prevalence or incidence of these atypical symptoms following concussion. The lack of standardized assessment tools for many of these atypical symptoms also poses a challenge in comparing outcomes across studies. Furthermore, the limited long-term follow-up in many cases makes it difficult to determine the true course and prognosis of these symptoms. Lastly, the complex interplay between brain injury, pre-existing conditions, and psychosocial factors in many of these cases makes it challenging to definitively attribute the observed symptoms solely to the concussion. The existing case literature lacks sufficient detail to draw meaningful conclusions about causation, and we therefore did not attempt to propose theoretical mechanisms underlying these functional disorders. Psychological factors, such as fear-avoidance behavior (Picon et al., 2021) and high levels of anxiety and depression (Jobin et al., 2023), have been strongly linked to functional neurological symptoms following a concussion, with anxiety scores particularly associated with unexpected somatic complaints (Picon et al., 2021). Additionally, individuals with pre-existing psychiatric conditions, including mood and anxiety disorders (Polich et al., 2024), or developmental challenges, such as attention deficits, are at greater risk for developing FND symptoms post-concussion (June et al., 2024). Future research would benefit from larger sample sizes, standardized assessment protocols, and advanced neuroimaging techniques to address these limitations. Additionally, targeted studies examining potential neurobiological, psychological, and social mechanisms would complement this descriptive review and provide a more comprehensive understanding of these atypical post-concussion presentations.

## CONCLUSION

This review highlights the diverse and complex nature of atypical concussive symptoms described in studies with detailed, patient-level data, encompassing visual, psychiatric, hearing, speech, and gait changes. The cases presented underscore the importance of comprehensive evaluation and individualized treatment approaches for patients experiencing these unusual symptoms. While many of these atypical presentations resolve spontaneously or with targeted interventions, some persist and require long-term management. The findings emphasize the need for clinicians to consider both organic and functional etiologies when assessing post-concussive symptoms. Future research should focus on developing standardized assessment tools, exploring the mechanisms underlying these atypical presentations, and establishing evidence-based treatment protocols. By improving the understanding of these less common manifestations, clinicians can enhance patient care and outcomes in the complex landscape of post-concussive disorders.

## Supplementary Material

Supplementary_File_Atypical_Review_acaf051
