# Supplementary material for: Atypical Symptoms Following Concussion: A Comprehensive Review of Functional Deficits
Source: Arch Clin Neuropsychol. 2025 Jun 9;40(7):1415–26. doi: 10.1093/arclin/acaf051 (PMC12540245; doi:10.1093/arclin/acaf051)
Supplement: Supplementary_File_Atypical_Review_acaf051 [file supplementary_file_atypical_review_acaf051.docx]

**Atypical Symptoms Following Concussion: A Comprehensive Review of Functional Deficits**

**Supplementary File**

Below is the complete search term for the systematic review.

("Brain Concussion"[Mesh] OR brain commotion[tiab] OR commotio cerebri[tiab] OR concuss*[tiab] OR mild brain injur*[tiab] OR mild brain trauma*[tiab] OR mild head injur*[tiab] OR mild head trauma*[tiab] OR mild TBI*[tiab] OR mild traumatic brain injur*[tiab] OR mild traumatic cerebral injur*[tiab] OR mild traumatic head injur*[tiab] OR minor brain injur*[tiab] OR minor brain trauma*[tiab] OR minor head injur*[tiab] OR minor head trauma*[tiab] OR minor TBI*[tiab] OR minor traumatic brain injur*[tiab] OR mTBI*[tiab] OR pmTBI*[tiab] OR postconcuss*[tiab]) AND ("Anomia"[Mesh] OR "Aphasia"[Mesh] OR "Aphonia"[Mesh] OR "Asthenia"[Mesh] OR "Blindness"[Mesh] OR "Cataplexy"[Mesh] OR "Complex Regional Pain Syndromes"[Mesh] OR "Conversion Disorder"[Mesh] OR "Deglutition"[Mesh] OR "Deglutition Disorders"[tiab] OR "Dissociative Disorders"[Mesh] OR "Dystonia"[Mesh] OR "Dystonic Disorders"[Mesh] OR "Fibromyalgia"[Mesh] OR "Hallucinations"[Mesh] OR "Hysteria"[Mesh] OR "Irritable Bowel Syndrome"[Mesh] OR "Medically Unexplained Symptoms"[Mesh] OR "Muscle Weakness"[Mesh] OR "Neurasthenia"[Mesh] OR "Paralysis"[Mesh] OR "Psychophysiologic Disorders"[Mesh] OR "Postural Orthostatic Tachycardia Syndrome"[Mesh] OR "Prosopagnosia"[Mesh] OR "Psychogenic Nonepileptic Seizures"[Mesh] OR "Psychosomatic Medicine"[Mesh] OR "Somatoform Disorders"[Mesh] OR "Spinothalamic Tracts"[Mesh] OR "Stuttering"[Mesh] OR "Tic Disorders"[Mesh] OR "Tics"[Mesh] OR "Tourette Syndrome"[Mesh] OR absence of vision[tiab] OR absence of visual[tiab] OR absence of vocal*[tiab] OR absence of voice*[tiab] OR absent vision[tiab] OR absent visual[tiab] OR absent vocal*[tiab] OR absent voice*[tiab] OR adynamia[tiab] OR agnosia[tiab] OR agnosias[tiab] OR Akureyri[tiab] OR alogia[tiab] OR amauroses[tiab] OR amaurosis[tiab] OR anepia[tiab] OR anomia[tiab] OR anomias[tiab] OR anomic[tiab] OR anomy[tiab] OR aphantasia[tiab] OR aphasia[tiab] OR aphasias[tiab] OR aphonia[tiab] OR aphonias[tiab] OR arm weak*[tiab] OR astasia abasia[tiab] OR asthaenia[tiab] OR asthenia[tiab] OR asthenias[tiab] OR asthenic[tiab] OR blindness[tiab] OR blindnesses[tiab] OR bodily distress disorder[tiab] OR bodily distress disorders[tiab] OR cataplectic[tiab] OR cataplecticus[tiab] OR cataplex*[tiab] OR chronic fatigue[tiab] OR chronic subjective dizziness[tiab] OR colon spasm[tiab] OR colon spasms[tiab] OR colonic spasm[tiab] OR colonic spasms[tiab] OR conversion disorder[tiab] OR conversion neuroses[tiab] OR conversion neurosis[tiab] OR conversion reaction[tiab] OR conversion reactions[tiab] OR conversion seizure[tiab] OR conversion seizures[tiab] OR conversion syndrome[tiab] OR deglutition[tiab] OR deglutitions[tiab] OR dissociation[tiab] OR dissociative[tiab] OR dysfluen*[tiab] OR dysnomia[tiab] OR dysnomias[tiab] OR dysnomic[tiab] OR dysphagia[tiab] OR dysphagias[tiab] OR dysphasia[tiab] OR dysphasias[tiab] OR dystonia[tiab] OR dystonias[tiab] OR dystonic[tiab] OR epidemic neuromyasthenia[tiab] OR facial twitch*[tiab] OR fatigue syndrome[tiab] OR fatigue syndromes[tiab] OR fatigued musc*[tiab] OR fibro-myalg*[tiab] OR fibromyalg*[tiab] OR fibrositic[tiab] OR fibrositis[tiab] OR fluency disorder[tiab] OR fluency disorders[tiab] OR functional colon*[tiab] OR functional dizziness[tiab] OR functional movement disorder[tiab] OR functional movement disorders[tiab] OR functional neurologic*[tiab] OR functional seizure[tiab] OR functional seizures[tiab] OR habit spasm[tiab] OR habit spasms[tiab] OR habitual spasm[tiab] OR hallucinat*[tiab] OR heightened olfaction[tiab] OR heightened olfactory[tiab] OR hyperolfaction[tiab] OR hyperosmia[tiab] OR hyperosmic[tiab] OR hysteria*[tiab] OR hysterical*[tiab] OR Iceland disease[tiab] OR insufficient musc*[tiab] OR irritable bowel syndrome[tiab] OR irritable bowel syndromes[tiab] OR irritable colon*[tiab] OR legasthen*[tiab] OR Lichtheim*[tiab] OR limb weak*[tiab] OR logagnosia[tiab] OR loss of motor[tiab] OR loss of muscle strength[tiab] OR loss of muscular strength[tiab] OR loss of phonation[tiab] OR loss of sense*[tiab] OR loss of sensory[tiab] OR loss of strength[tiab] OR loss of vision[tiab] OR loss of visual[tiab] OR loss of vocal*[tiab] OR loss of voice*[tiab] OR lost sensory[tiab] OR lost strength[tiab] OR lost vision[tiab] OR lost visual[tiab] OR lost vocal*[tiab] OR lost voice*[tiab] OR medical unexplain*[tiab] OR medically unexplain*[tiab] OR motor loss[tiab] OR motor losses[tiab] OR mucomembraneous colitis[tiab] OR mucomembranous colitis[tiab] OR mucous colitides[tiab] OR mucous colitis[tiab] OR muscle fatigue[tiab] OR muscle insufficien*[tiab] OR muscle strength loss[tiab] OR muscle weak*[tiab] OR muscular fatigue[tiab] OR muscular rheumatism[tiab] OR muscular insufficien*[tiab] OR muscular strength loss[tiab] OR muscular weak*[tiab] OR myalgic encephalomyelitis[tiab] OR myodystonia[tiab] OR myodystonic[tiab] OR myodystony[tiab] OR myoparalysis[tiab] OR neurasthen*[tiab] OR nervous twitch*[tiab] OR neuroasthen*[tiab] OR neurologic functional[tiab] OR neurological functional[tiab] OR neuromuscular fatigue[tiab] OR neuromuscular insufficien*[tiab] OR non epileptic[tiab] OR non organic*[tiab] OR nonepileptic[tiab] OR nonorganic*[tiab] OR olfactory hyperaesthesia[tiab] OR olfactory hyperesthesia[tiab] OR orthostatic tachycardia[tiab] OR pain disorder[tiab] OR pain disorders[tiab] OR palsies[tiab] OR palsy[tiab] OR paralyses[tiab] OR paralysis[tiab] OR paralytic[tiab] OR paralytics[tiab] OR paroxysmal event[tiab] OR paroxysmal events[tiab] OR persistent perceptual postural dizziness[tiab] OR persistent positional perceptual dizziness[tiab] OR persistent postural perceptual dizziness[tiab] OR phobic postural vertigo[tiab] OR plegia[tiab] OR plegias[tiab] OR plegic[tiab] OR postural phobic vertigo[tiab] OR positional tachycardia[tiab] OR postural tachycardia[tiab] OR POTS[tiab] OR PPPD[tiab] OR prosapagnosia[tiab] OR prosopagnosia[tiab] OR prosopagnosias[tiab] OR prosophthalmia[tiab] OR pseudo-seizure[tiab] OR pseudo-seizures[tiab] OR pseudoseizure[tiab] OR pseudoseizures[tiab] OR psycho-autonomic[tiab] OR psycho-neurotic[tiab] OR psycho-physical*[tiab] OR psycho-physiologic*[tiab] OR psycho-somatic*[tiab] OR psycho-somatolog*[tiab] OR psychoautonomic[tiab] OR psychogenic[tiab] OR psychoneurotic[tiab] OR psychophysical*[tiab] OR psychophysiologic*[tiab] OR psychosomatic*[tiab] OR psychosomatolog*[tiab] OR psychosomatosis[tiab] OR region pain*[tiab] OR regional body pain*[tiab] OR regional pain*[tiab] OR royal free disease[tiab] OR sensory loss[tiab] OR sensory losses[tiab] OR smell hypersensitiv*[tiab] OR somatic physical symptom*[tiab] OR somatic symptom*[tiab] OR somatization[tiab] OR somato-psych*[tiab] OR somatoform[tiab] OR somatopsych*[tiab] OR spastic colitis[tiab] OR spastic colon*[tiab] OR speech disorder[tiab] OR speech disorders[tiab] OR speech disturbance[tiab] OR speech disturbances[tiab] OR spinal thalamic*[tiab] OR spinal thalamus[tiab] OR spino-thalamic*[tiab] OR spino-thalamus[tiab] OR spinothalamic*[tiab] OR spinothalamus[tiab] OR stammer*[tiab] OR strength loss[tiab] OR strength losses[tiab] OR stutter*[tiab] OR super smell*[tiab] OR supersmell*[tiab] OR swallow*[tiab] OR systemic exertion intolerance[tiab] OR tic[tiab] OR tics[tiab] OR Tourette*[tiab] OR unexpected neurologic*[tiab] OR unexpected physical symptom*[tiab] OR unexpected symptom*[tiab] OR unexpected syndrome*[tiab] OR unexplainable neurologic*[tiab] OR unexplainable physical symptom*[tiab] OR unexplainable symptom*[tiab] OR unexplainable syndrome*[tiab] OR unexplained neurologic*[tiab] OR unexplained physical symptom*[tiab] OR unexplained symptom*[tiab] OR unexplained syndrome*[tiab] OR unstable colon*[tiab] OR vision loss[tiab] OR vision losses[tiab] OR visual loss[tiab] OR visual losses[tiab] OR visual vertigo[tiab] OR visually-induced vertigo[tiab] OR voice absence[tiab] OR weak arm[tiab] OR weak arms[tiab] OR weak limb[tiab] OR weak limbs[tiab] OR weak musc*[tiab] OR weakened limb[tiab] OR weakened limbs[tiab] OR weakened musc*[tiab] OR word deaf*[tiab] OR word finding[tiab] OR yuppie flu[tiab] OR "functional disorder"[tiab:~3] OR "functional disorders"[tiab:~3] OR "functional symptom"[tiab:~3] OR "functional symptoms"[tiab:~3] OR "functional syndrome"[tiab:~3] OR "functional syndromes"[tiab:~3])
